# ﻿Two new species of *Tmethypocoelis* Koelbel, 1897 (Decapoda, Brachyura, Dotillidae) from Sulawesi, Indonesia

**DOI:** 10.3897/zookeys.1156.98930

**Published:** 2023-03-30

**Authors:** Dewi Citra Murniati, Akira Asakura, Peter J. F. Davie

**Affiliations:** 1 Research Center for Biosystematics and Evolution, National Research and Innovation Agency (BRIN), Jl. Raya Jakarta Bogor Km 46, Cibinong, Bogor, Indonesia Research Center for Biosystematics and Evolution, National Research and Innovation Agency (BRIN) Bogor Indonesia; 2 Department of Zoology, Division of Biological Science, Graduate School of Science, Kyoto University, 606-8501, Yoshida-honmachi, Sakyo-ku, Kyoto-shi, Japan Kyoto University Kyoto-shi Japan; 3 Seto Marine Biological Laboratory, Field Science Education and Research Center, Kyoto University, 459 Shirahama, Nishimuro, Wakayama 649-2211, Japan Kyoto University Wakayama Japan; 4 Queensland Museum, PO Box 3300, South Brisbane, Qld 4101, Australia Queensland Museum Brisbane Australia

**Keywords:** Biogeography, Celebes, dotillid crabs, gastric mill, morphology

## Abstract

*Tmethypocoelis* Koelbel, 1897, is a central Indo-West Pacific genus of small intertidal, soft sediment dotillid crabs that includes five recognised species. Two new species, *Tmethypocoelissimplex***sp. nov.** and *T.celebensis***sp. nov.**, are here described from Sulawesi, Indonesia. *Tmethypocoelissimplex***sp. nov.** is found on the west coast of Central Sulawesi, while *T.celebensis***sp. nov.** occurs in the north-eastern part of Sulawesi. Both new species differ from each other and known congeners by the male cheliped, male pleon, and male first gonopod characters. The differences in gastric mill morphology further confirm the two species as new. The distinct water current patterns in the Makassar Strait and the Maluku Channel might have contributed to the evolution of these two sibling species.

## ﻿Introduction

Crabs of the dotillid genus, *Tmethypocoelis* Koelbel, 1897, are small and found on intertidal mudflats and estuarine mud or sandy-mud banks, often extending into low salinity (Dutreix 1992). *Tmethypocoelis* is unique among confamilials by having a long narrow apical styliform projection on the eyestalks that extends beyond the cornea (Davie 1990).

The first species to be described was *Tmethypocoelisceratophora* (Koelbel, 1897) from Hong Kong. Although it was initially placed in *Dioxippe* de Man, 1888, Koelbel (1897, 1898) remarked that its unique characters deserved the recognition of a new subgenus Dioxippe (Tmethypocoelis) Koelbel, 1897. Five species are currently known in *Tmethypocoelis: T.ceratophora* (Koelbel, 1897) from China; *T.koelbeli* Davie, 1990, from Northern Territory, Australia (Davie 1990; Davie and Kosuge 1995); *T.odontodactylus* Davie, 1990, from Madang, Papua New Guinea (Davie 1990; Davie and Kosuge 1995); *T.choreutes* Davie & Kosuge, 1995, from Japan (Davie and Kosuge 1995); *T.liki* Murniati, Asakura, Nugroho, Hernawan & Dharmawan, 2022, from Papua, eastern Indonesia (Murniati et al. 2022). The previous records of *T.ceratophora* from Japan and Indonesia (Dutreix 1992; Huang et al. 1992; Murniati 2015) would appear to be misidentifications, and the Japanese specimens have since been described as *T.choreutes* Davie & Kosuge, 1995. The specimen first recorded from Lombok, Indonesia, is also likely to be a new species and will be further discussed in a subsequent paper with other potential new species from the Indonesian region following ongoing revisionary work on this genus.

Fieldwork by the first author to investigate the systematics of the Dotillidae Stimpson, 1858, of Indonesia has resulted in the discovery of populations of two species occurring on opposite coasts of Sulawesi Island, Indonesia, here described as both new to science and compared with the previously known species of *Tmethypocoelis*.

## ﻿Materials and methods

### ﻿Sampling

**Figure 1.**
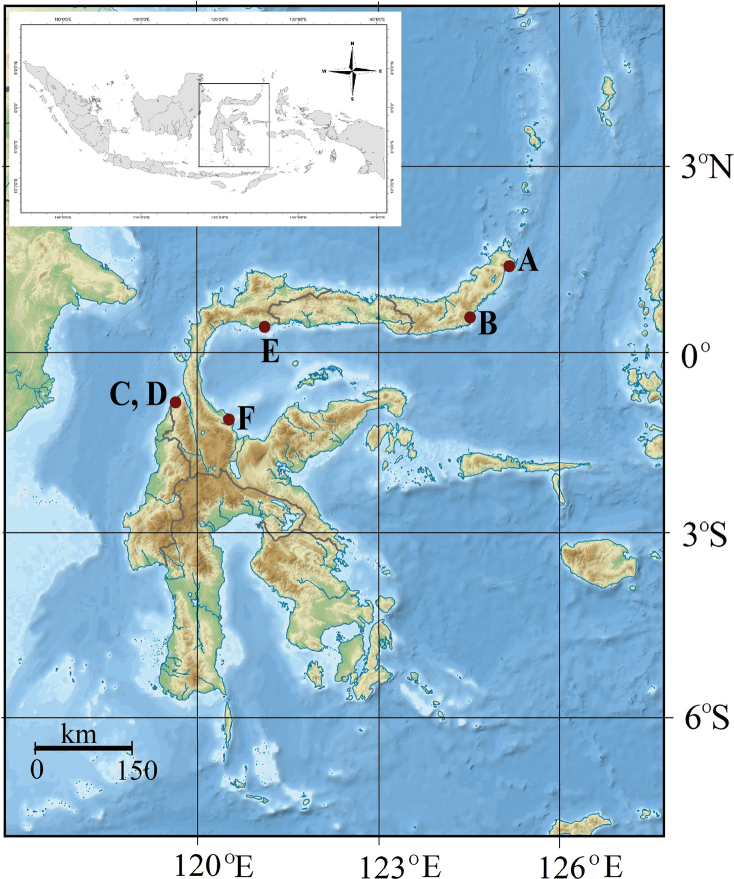
Map of Sulawesi. Sampling stations **A** Moletang Beach, Kema Tiga,North Minahasa, North Sulawesi **B** Iyok Beach, East Bolang Mogondow, North Sulawesi **C** Towale, CentralBanawa District, Donggala, Central Sulawesi **D** Tosale, Central Banawa District, Donggala, Central Sulawesi **E** Tuladenggi Sibatang, Parigi Moutong, Central Sulawesi **F** Maleyali, Sausu, Parigi Moutong, Central Sulawesi (derived from Wikipedia 2022).

Fieldwork to Sulawesi Island was conducted in September 2020 and June 2021 at six sampling estuarine sites: Moletang River (estuary), Kema Tiga, North Minahasa, North Sulawesi, 1°21'59.6"N, 125°04'38.9"E; Iyok Beach, East Bolang Mongondow, North Sulawesi, 0°35'06.0"N, 124°31'58.6"E; Towale River, Central Banawa District, Donggala, Central Sulawesi, 0°43'29.3"S, 119°40'43.9"E; Tosale, Banawa District, Donggala, Central Sulawesi, 0°45'57.1"S, 119°40'58.4"E; Tuladenggi Sibatang, Parigi Moutong, Central Sulawesi, 0°24'41.0"N, 121°07'43.9"E; Maleyali, Sausu, Parigi Moutong, Central Sulawesi, 1°05'31.0"S, 120°33'39.6"E (Fig. 1). The crab specimens were collected by hand during diurnal low tide periods. All specimens were preserved in 90% ethanol.

### ﻿Morphology

The crabs were observed, measured, and photographed using either a stereo microscope (Olympus SZ) connected with a digital camera (Olympus E-330), or a Leica Z6 microscope connected to a computer using LAS Core v. 4.13 software.

Measurements in millimeters (mm) were of carapace width (**cw**, measured across at the widest point) and carapace length (**cl**, measured from the mid-front to the mid-posterior margin). Smaller specimens and body parts were examined under a Nikon SMZ 800 stereo Microscope equipped with a camera lucida drawing tube. Drawings were made by hand and enhanced using a Wacom drawing pad and Adobe Illustrator CC2015 software.

Morphological terminology for the teeth of the gastric mill follows Davie et al. (2015). The G1 and teeth on the gastric mill were removed, fixed in glutaraldehyde and cacodylate buffer, serially dehydrated in ethanol (50%, 70%, 85%, 90%, 100%), and vacuum-dried using TITEC VC-96N for 10 minutes. Each prepared sample was then mounted on a specimen stub and coated with gold at 5–8 mA for 5 min using ion coater (Dewi and Purwaningsih 2020). The detailed photos of the teeth plate of the gastric mills and gonopods were captured using a scanning electron microscope (SEM), JEOL JSM-IT 200, at an accelerating voltage of 5 kV. The photographs of the teeth plate are presented with posterior portion upper-most.

Specimens have been deposited in the following repositories: Directorate of Scientific Collection Management, BRIN, Cibinong, Bogor, Indonesia (**MZB**); Lee Kong Chian Natural History Museum, National University of Singapore, Singapore (**ZRC**); Osaka Museum of Natural History, Japan (**OMNH**); Naturalis Biodiversity Center, The Netherlands (**RMNH**); and Queensland Museum, Australia (**QM**).

### ﻿Abbreviations

**Pl** pleonite;

**P** pereiopod;

**G1** male first gonopod;

**ovig** ovigerous.

## ﻿Taxonomy

### ﻿ Dotillidae

Taxon classificationAnimaliaDecapodaDotillidae

Stimpson, 1858

A1B6FF7E-9129-5EA7-8D64-56A4F758088B

Tmethypocoelis Koelbel, 1897.
Dioxippe (Tmethypocoelis) Koelbel, 1897: 715; 1898: 574.
Tmethypocoelis – Shen 1935: 33. — Sakai 1939: 643; 1976: 625. — Davie 1990: 463; 2002: 347. — Dai et al. 1986: 451. — Dai and Yang 1991: 495. — Huang et al. 1992: 150 (key), 154. — Wada 1995: 415 (key). — Davie and Kosuge 1995: 208. — Ng et al. 2008: 235. — Shih et al. 2015: 61.

#### Type species.

Dioxippe (Tmethypocoelis) ceratophora Koelbel, 1897, by original designation, subsequently elevated to generic status by Shen (1935); gender feminine.

#### Remarks.

The genus name *Dioxippe* de Man 1888, to which the type species was originally placed, is pre-occupied by *Dioxippe* Thomson, 1860 [Coleoptera]; and therefore, a replacement name, *Tympanomerus*, was proposed by Rathbun (1897), and subsequently used by Tesch (1918) in his account of *Tympanomerusceratophora*. However, *Tympanomerus* Rathbun, 1897, is itself currently considered a junior synonym of *Ilyoplax* Stimpson, 1858. Shen (1935) was the first to formally elevate *Tmethypocoelis* to full generic rank.

The year of publication of *Tmethypocoelisceratophora* has usually been wrongly attributed to Koelbel (1898); the 1898 paper is actually a German translation of Koelbel’s original paper published in 1897 in Hungarian. The name should thus be cited as *Tmethypocoelisceratophora* (Koelbel, 1897).

### ﻿ Tmethypocoelis simplex sp. nov.

Taxon classificationAnimaliaDecapodaDotillidae

128BBC35-A992-5CD6-8424-A180D14CB963

https://zoobank.org/F7FC7DED-1435-42E8-8428-5B38FF061959

Figs 2 , 3 , 4 , 5 , 6 , 7 , 8 , 9 , 17A, C

#### Material examined.

***Holotype*. Indonesia** • 1 ♂ (7.7 × 4.4 mm); Tosale, Banawa District, Donggala, Central Sulawesi; 0°45'57.1"S, 119°40'58.4"E; 17 Sep. 2020; coll. DC. Murniati, D. Permatasari, Hairul, A. Padju; MZB.Cru.5573.

***Paratypes*. Indonesia** • 12 ♂ (4.0 × 2.5 – 7.9 × 4.6), 6 ♀ (5.8 × 3.6 – 6.5 × 4.0 mm); Towale River, Central Banawa District, Donggala, Central Sulawesi; 0°43'29.3"S, 119°40'43.9"E; 17 Sep. 2020; coll. DC. Murniati, D. Permatasari, Hairul, A. Padju; MZB.Cru.5182 • 15 ♂ (4.1 × 2.6 – 7.7 × 4.4 mm), 4 ♀ (3.8 × 2.6 – 6.1 × 3.9 mm); same data as for holotype; MZB.Cru.5183 • 4 ♂ (4.7 × 3.0 – 5.3 × 3.3 mm), 4 ♀ (4.0 × 2.5 – 5.6 × 3.3 mm); Towale River, Central Banawa District, Donggala, Central Sulawesi; 0°43'29.3"S, 119°40'43.9"E; 17 Sep. 2020; coll. DC. Murniati, D. Permatasari, Hairul, A. Padju; ZRC 2023.0055 • 4 ♂ (4.0 × 2.5 – 5.1 × 3.0 mm); Towale River, Central Banawa District, Donggala, Central Sulawesi; 0°43'29.3"S, 119°40'43.9"E; 17 Sep. 2020; coll. DC. Murniati, D. Permatasari, Hairul, A. Padju; OMNH-Ar.12758–12761 • 4 ♀ (5.8 × 3.5 – 6.6 × 4.0 mm); Towale River, Central Banawa District, Donggala, Central Sulawesi; 0°43'29.3"S, 119°40'43.9"E; 17 Sep. 2020; coll. DC. Murniati, D. Permatasari, Hairul, A. Padju; OMNH-Ar.12762–12765 • 6 ♂ (5.5 × 3.4 – 6.7 × 4.0 mm), 5 ♀ (5.0 × 3.1 – 5.8 × 3.5 mm); Towale River, Central Banawa District, Donggala, Central Sulawesi; 0°43'29.3"S, 119°40'43.9"E; 17 Sep. 2020; coll. DC. Murniati, D. Permatasari, Hairul, A. Padju; RMNH.CRUS.D.58046 • 3 ♂ (6.5 × 3.7 mm – 7.5 × 4.3 mm); Tosale, Banawa District, Donggala, Central Sulawesi; 0°45'57.1"S, 119°40'58.4"E; 17 Sep. 2020; coll. DC. Murniati, D. Permatasari, Hairul, A. Padju; QM W29642.

#### Comparative material.

*Tmethypocoelisliki* Murniati, Asakura, Nugroho, Hernawan & Dharmawan, 2022: Indonesia • paratypes 5 ♂ (5.3 × 3.1 mm – 5.5 × 3.2 mm); Liki Village, Sarmi District, Sarmi Municipality, Liki Island, Papua Province; 01°37'25.29"S, 138°44'26.54"E; 21 Nov. 2018; coll. DC. Murniati; MZB.Cru.5012.

#### Diagnosis.

**Figure 2.**
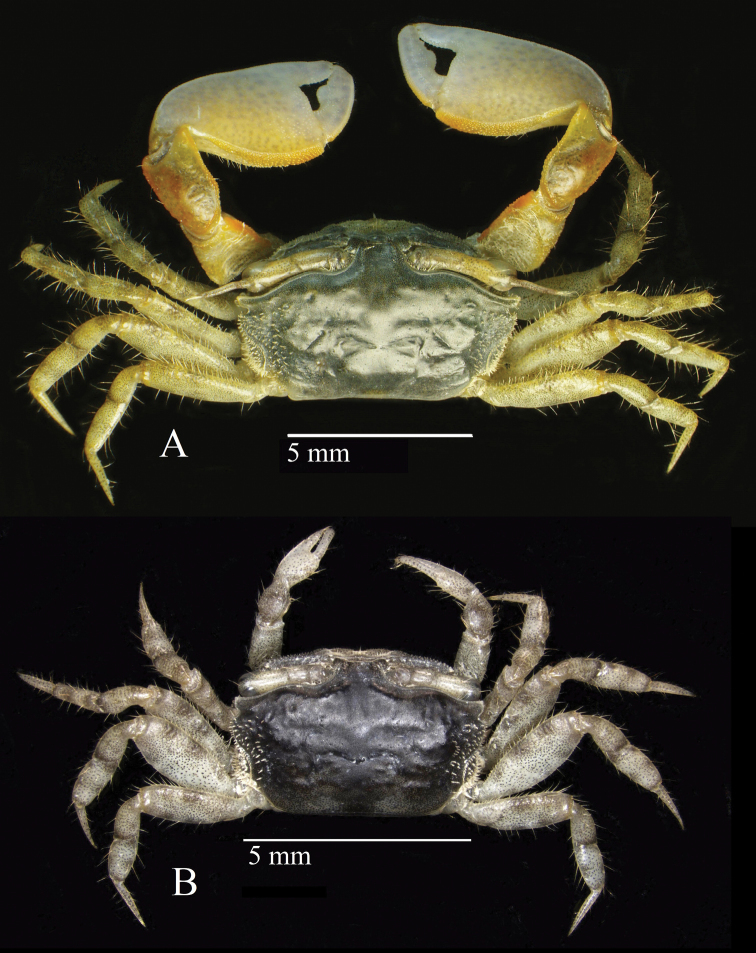
Dorsal habitus of *Tmethypocoelissimplex* sp. nov. from Towale River, Central Banawa District, Donggala, Central Sulawesi **A** holotype, male (7.7 × 4.4 mm) (MZB.Cru.5573) **B** paratype, female (5.8 × 3.6 mm) (MZB.Cru. 5182).

Carapace pentagonal, ca. 1.7× as wide as long (Fig. 2A). Branchial regions sloping; protobranchial, mesobranchial and metabranchial regions well-defined. Sub-branchial region bulging, bearing regular setae and tubercles. Posterior margin slightly concave, ca. 0.53 distance between exorbital angles. Exorbital angle triangular, acute, directed forward (Fig. 4A). Second anterolateral tooth of carapace slightly acute, slightly shorter than exorbital angle. Male pleon ca. 2× as long as wide (Fig. 4E). Male chelipeds long (Fig. 5); palm bulky, ca. 1.4× as long as wide; fingers shorter than palm; pollex short, triangular, cutting margin gently convex over entire length, without differentiated tooth or lobe; dactylus cutting margin evenly dentate, one enlarged wide convex tooth over proximal half, upper margin with one median row of granules in simple row, narrower distally (Fig. 5O). G1 long, curved, conspicuously slender; apical portion forming two poorly defined lobes, with three conspicuously curved setae on outer margin becoming slightly longer distally, two or three long setae apically, and four short setae on inner margin (Fig. 8A–E).

#### Description.

**Figure 3.**
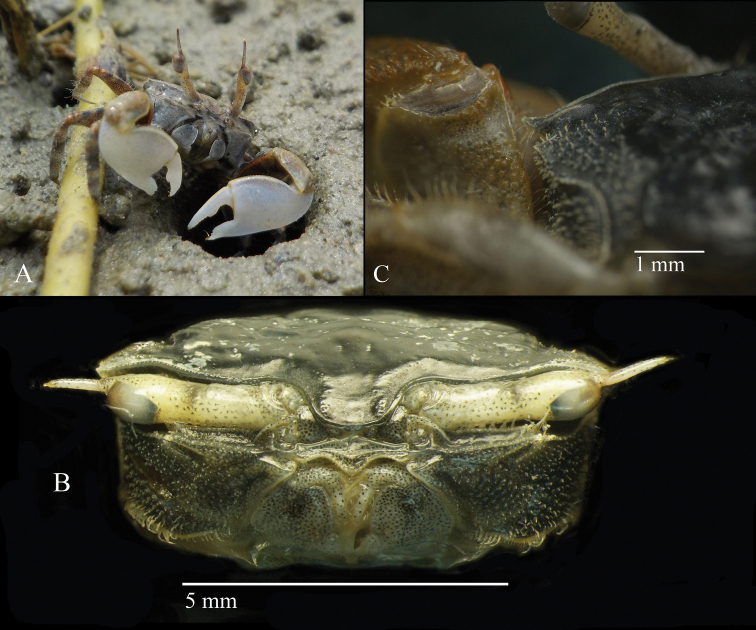
*Tmethypocoelissimplex* sp. nov. Holotype, male (7.7 × 4.4 mm) (MZB.Cru.5573), Towale River, Central Banawa District, Donggala, Central Sulawesi **A** in-situ with live coloration **B** front area **C** merus of left cheliped held against external orbital angle.

**Figure 4.**
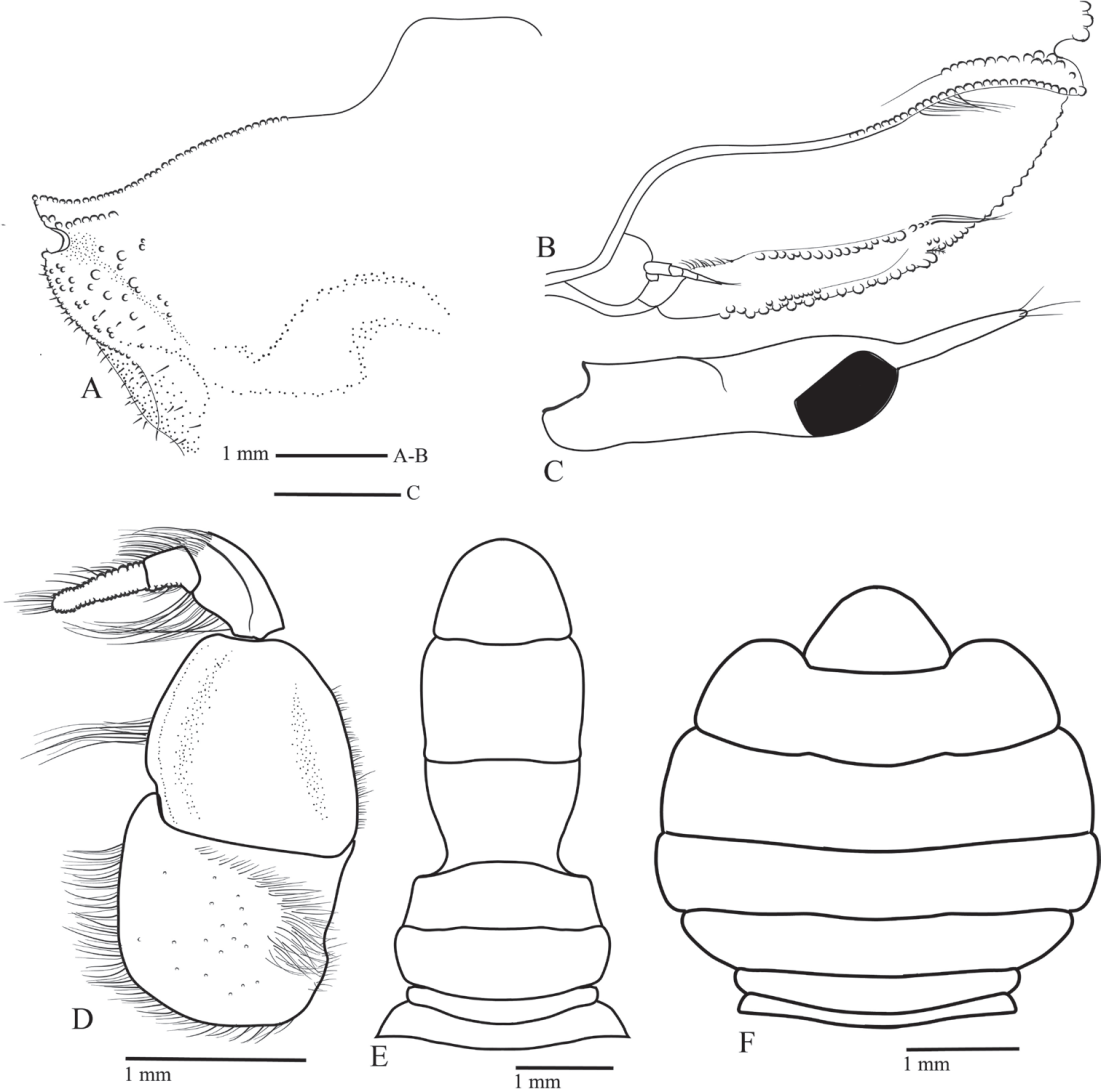
*Tmethypocoelissimplex* sp. nov. Holotype, male (7.7 × 4.4 mm) (MZB.Cru.5573), Towale River, Central Banawa District, Donggala, Central Sulawesi **A** external orbital angle **B** orbit area **C** eyestalk **D** third maxilliped **E** pleon. Paratype, female (5.8 × 3.6 mm) (MZB.Cru. 5182), Towale River, Central Banawa District, Donggala, Central Sulawesi **F** pleon.

Carapace (Fig. 2A, B) pentagonal, weakly convex along mid-dorsal line, weakly convex laterally, ca. 1.7× as wide as long. Dorsal surface smooth, lateral portion with granules, regions semi-defined; epigastric lobe poorly defined. Cervical grooves well-marked. Cardiac region with slight central depression. Branchial regions sloping; protobranchial, mesobranchial and metabranchial regions well-defined. Sub-branchial region bulging, bearing regular setae and tubercles. Carapace widest between exorbital angles. Intestinal and branchial borders poorly defined. Lateral margin of carapace recurved, with row of tubercles and short stout setae. Posterior margin weakly concave, ca. 0.53 distance between exorbital angles; fine ridge parallel to posterior margin forming broad rim. Front with lateral border moderately converging, width at base ca. 0.24× distance between exorbital angles, ca. 0.21 at anterior margin; frontal angle rounded; anterior margin with small central blunt prominence (Fig. 3B). Exorbital angle triangular, acute, directed forward; anterior margin with microscopic tubercles, lateral margin glabrous; one short tubercular ridge parallel to supraorbital margin; posteriorly followed by broad U-shaped sinus. Second anterolateral tooth slightly acute, slightly shorter than exorbital angle. Posterolateral facet well-defined by crest originating anteriorly from base of exorbital angle (Fig. 4A). Supraorbital margin sinuous, sloping backward, microscopically tubercular. Infraorbital margin with medial notch; pterygostome with inwardly directed oblique channel; inner portion consisting of two granular ridges separated by shallow channel; upper ridge granular, with one row of setae; lower ridge granular, granules larger than those of upper ridge, without setation; outer portion granular, concave, ending in broad notch below exorbital angle (Fig. 4B).

Eyestalks long, not reaching exorbital angle, medial and distal diameters of similar width; projecting ocular style as long as cornea, tipped with setae; medial slope giving twisted appearance; cornea slightly bulging (Figs 2, 3B, 4C).

Third maxillipeds slightly vaulted, not completely covering buccal cavern. Ischium subquadrate; upper-mesial angle with wide triangular lobe; anterolateral angle narrow and triangular; mesial and lower margins with dense setae; lateral margin with setation medially; outer surface with oblique row of dense long setae, scattered granules distributed unevenly (Fig. 4D). Merus slightly larger than ischium; lateral margin convex, narrower distally, covered with short setae; mesial margin straight, with long setae; outer surface covered with scattered short setae (Fig. 4D). Carpus trihedral in cross section, mesial margin with dense long setae (Fig. 4D). Propodus short, margins tubercular and covered with dense setae (Fig. 4D). Dactylus slender, long, twice as long as propodus, margins tubercular, with long dense setae (Fig. 4D).

Male pleon (Fig. 4E) ca. 2× longer than wide; noticeably constricted at base of pleonite 5 (Pl5). Pl1 trapezoidal, narrow, ca. 9.5× wider than long; anterior margin ca. 0.7× as long as posterior margin; ca. 1.3× wider than Pl2. Pl2 ca. 7.5× as wide as long. Pl3 ca. 3.0× wider than long, anterior margin nearly straight, posterior margin convex. Pl4 ca. 2.9× as wide as long, widest proximally, narrowing distally, distolateral angle pointed. Pl5 ca. 1.4× wider than long (widest distally), markedly constricted at base. Pl6 ca. 1.4× as wide as long; widest sub-distally; 1.1× longer than Pl5; lateral margins subparallel, slightly concave. Male telson rounded, ca. 1.4× wider than long (Fig. 4E).

Female pleon conspicuously broad (Fig. 4F). Pl1 shortest; Pl2 distinctly longer, as wide as Pl1; Pl3 trapezoidal, longer than Pl2; Pl4 rectangular, slightly longer than Pl3, lateral margins convex; Pl5 longer than Pl4; Pl6 distinctly longest. Female telson triangular (Fig. 4F).

**Figure 5.**
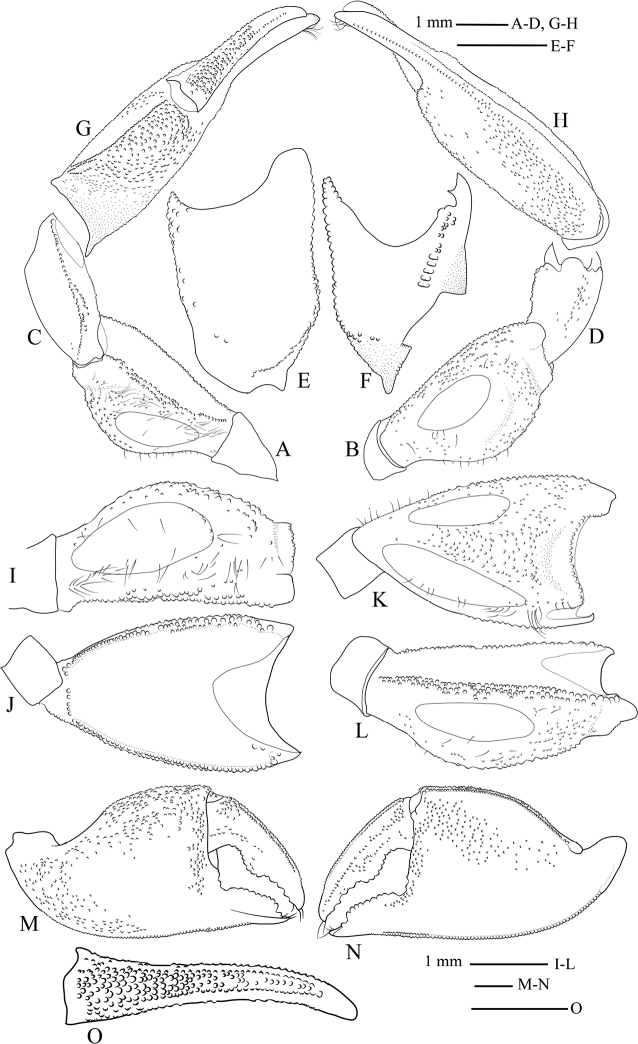
*Tmethypocoelissimplex* sp. nov. Holotype, male (7.7 × 4.4 mm) (MZB.Cru.5573), Towale River, Central Banawa District, Donggala, Central Sulawesi. Left cheliped **A** merus lower margin **B** merus outer surface. Carpus **C** outer surface **D** inner surface **E** upper surface **F** lower surface. Chela **G** upper margin **H** lower margin. Merus **I** upper surface **J** lower surface **K** upper margin **L** outer margin. Chela **M** inner surface **N** outer surface **O** dactylus upper margin.

Male chelipeds stout, long, equal. Merus cross-section triangular; standing higher than exorbital angle (Fig. 3C); lower margin covered with granules extending entire length, granulation branched sub-medially into two rows (Fig. 5A); upper margin narrower proximally, wider distally, with irregular rows of granules on distal half, proximal portion smooth (Fig. 5K); outer margin with a single row of granules extending whole length, granulation branched proximally (Fig. 5L); upper surface slightly convex, with ovate tympanum, microscopic granules outside tympanum, granulation mainly on distal portion, scattered setae (Fig. 5I); lower surface smooth, flattened, without tympanum (Fig. 5J); outer surface slightly convex, wider than upper surface, tympanum smaller than on upper surface, granules outside tympanum distributed evenly (Fig. 5B). Carpus shorter than merus, elongate, ca. 1.5× as long as wide; upper and lower margins tubercular (Fig. 5C, D); outer surface rectangular, convex, microscopic granules only (Fig. 5E); inner surface shiny, with one oblique row of granules (Fig. 5F). Palm bulky, ca. 1.4× wider than long; upper margin with one row of granules, distinct groove extending below granular rows forming clear granular string (Fig. 5G); lower margin granular, granulation branched into two rows medial to distal portion (Fig. 5H); inner surface irregularly granular, upper granulation extending over median portion, curved to sharply cut upper margin of outer surface and base of fingers, lower granulation extending near lower margin from proximal portion to base of pollex (Fig. 5M); outer surface distinctly granular over upper half to base of pollex, lower half smooth (Fig. 5N). Fingers shorter than palm, broadly gaping at base; curved inwards, expanded distally forming spooned-tip; cutting margins evenly serrated; inner margin at tip of both fingers with short row of stout setae. Pollex short, triangular, cutting margin evenly dentate; long flat enlarged dentate tooth over most of length, ca. 0.4× as wide as palm; inner surface smooth (Fig. 5M); outer surface granular proximally parallel to cutting margin (Fig. 5N); lower margin granular nearly whole length (Fig. 5H). Dactylus ca. 0.6× as wide as palm; cutting margin evenly dentate; one enlarged wide convex tooth over proximal half; inner surface granular from proximal to median portion near cutting margin, one clutch of granules proximally near upper margin (Fig. 5M); outer surface with 1 row of granules medially, densest on proximal portion of surface, granulation extending nearly entire length, irregular granulation near cutting margin, a single row of spaced tubercles medially, parallel to upper margin (Fig. 5N); upper margin with median row of granules, narrower distally matching shape of upper margin (Fig. 5O).

**Figure 6.**
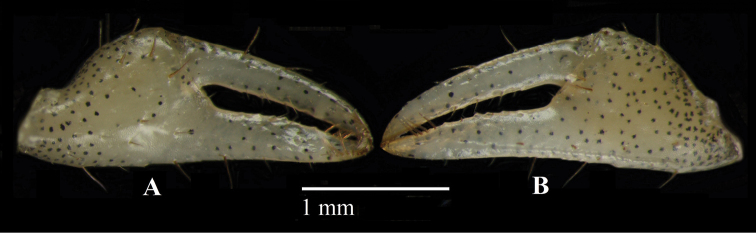
*Tmethypocoelissimplex* sp. nov. Paratype female (5.8 × 3.6 mm) (MZB.Cru. 5182), Towale river, Central Banawa District, Donggala, Central Sulawesi. Left chela **A** inner surface **B** outer surface.

Female chelipeds small, of typical dotillid type (Figs 2B, 6). Merus with ovate tympanum on upper and lower surfaces. Fingers longer than palm, spooned-tip (Fig. 6). Pollex outer surface with one tubercular ridge parallel to lower margin; lower margin entire; cutting margin with very low denticles. Dactylus cutting margin without denticles.

Pereiopods slender, elongate, P2–P5 similar; smooth ovate tympanum on anterior and posterior surfaces of meri. Tympani on posterior surfaces becoming progressively smaller from P2–P5. Dactyli nearly straight, pointed, shorter than propodi.

**Figure 7.**
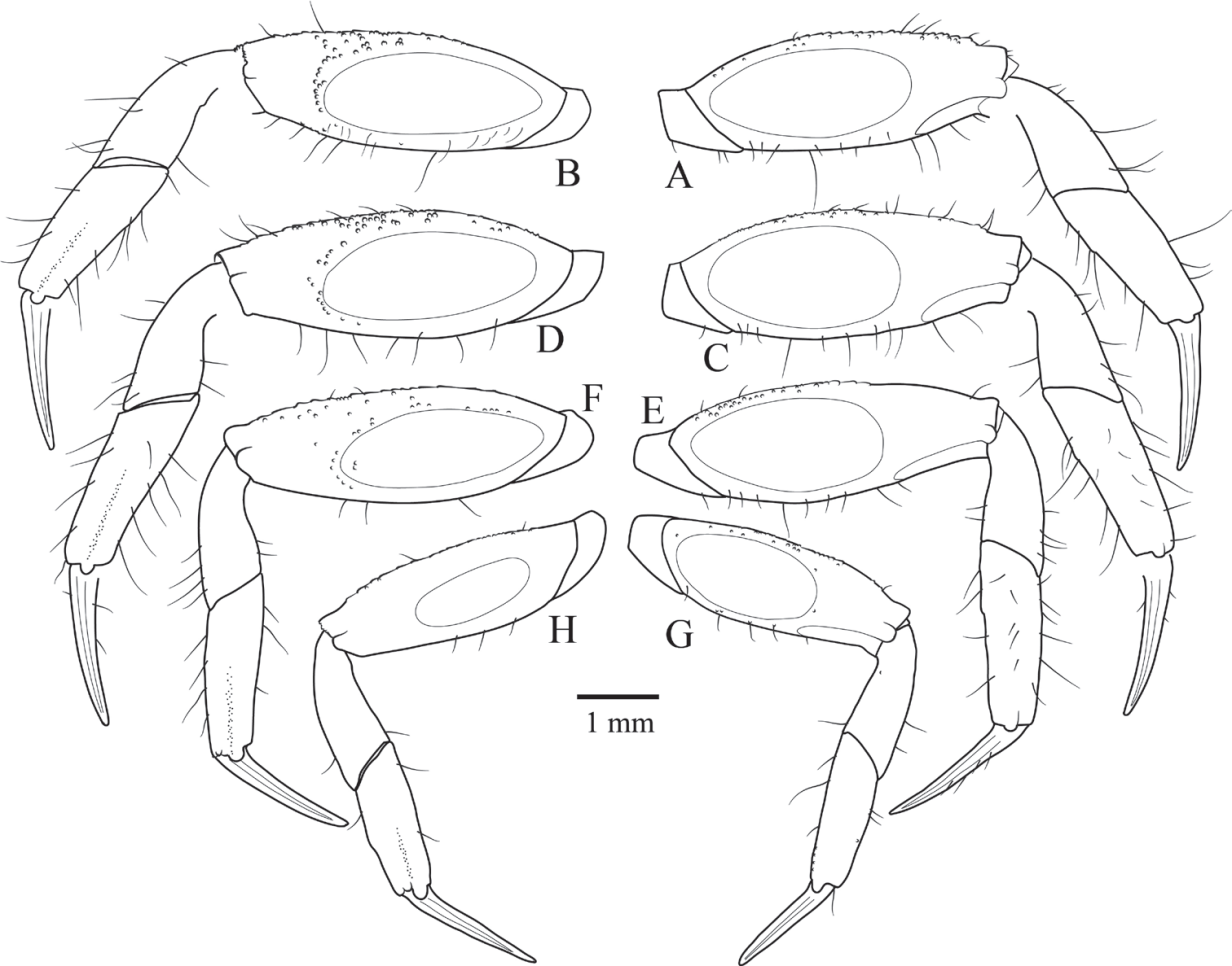
Left pereiopods of *Tmethypocoelissimplex* sp. nov. Holotype, male (7.7 × 4.4 mm) (MZB.Cru.5573), Towale River, Central Banawa District, Donggala, Central Sulawesi **A, B** P2 **C, D** P3 **E, F** P4; **G, H** P5. Right side, anterior surface; left side, posterior surface.

P2 (Fig. 7A, B) shorter than P3; merus ca. 2.84× longer than wide; anterior surface with scattered granules mainly near upper margin; posterior surface with sparse granules mainly near distal portion of tympanum; upper margin serrated, sparse long setae; lower margin smooth, sparse setae. Carpus subequal in length to propodus, surfaces smooth; margins smooth, sparse setae. Propodus with anterior and posterior surfaces bearing scattered granules; margins with sparse long setae.

P3 (Fig. 7C, D) longest; merus ca. 2.67× longer than wide; anterior surface with scattered granules, denser near upper margin; posterior surface with sparse granules denser distal to tympanum; upper margin serrated, sparse long setae; lower margin smooth, sparse setae. Carpus shorter than propodus, surfaces smooth; margins smooth, sparse setae. Propodus with anterior surface bearing sparse granules; posterior surface with sparse setae and granules; margins with sparse long setae.

P4 (Fig. 7E, F) merus ca. 2.78× longer than wide; anterior surface with scattered granules denser near upper margin; posterior surface sparsely granulate, denser towards upper margin; upper margin serrated, sparse long setae; lower margin smooth, sparse setae. Carpus shorter than propodus, surfaces smooth; margins smooth, sparse setae. Propodus with anterior surface with sparse setae and granules; posterior surface with granules; margins with sparse long setae.

P5 (Fig. 7G, H) shortest; merus ca. 2.71× longer than wide; anterior surface with scattered granules, denser near upper margin; posterior surface sparsely granulate, denser toward upper margin; upper margin serrated, sparse long setae; lower margin smooth, sparse setae. Carpus shorter than propodus, surfaces smooth; margins smooth, sparse setae. Propodus with anterior and posterior surfaces smooth; margins with sparse long setae.

**Figure 8.**
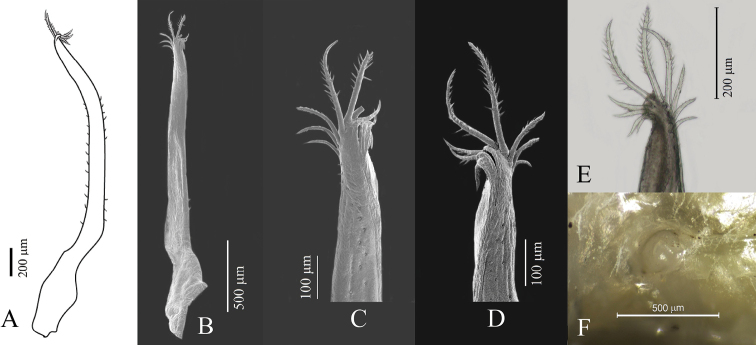
Reproductive organs of *Tmethypocoelissimplex* sp. nov. Paratype, males (**A–D** 4.1 × 2.6 mm**E** 6.9 × 4.0 mm) (MZB.Cru.5183), Tosale, Banawa District, Donggala, Central Sulawesi, left G1**A** dorsal view **B** apical (dorsal view) **C, D** apical (ventral view) **E** mesial view. Paratype female (5.8 × 3.6 mm) (MZB.Cru.5182), Towale river, Central Banawa District, Donggala, Central Sulawesi **F** vulva.

Reproductive organs. G1 (Fig. 8A–E) long, curved, very slender; sub-proximal bulge (Fig. 8A, B); apical portion forming two poorly defined lobes, with three conspicuous curved setae on outer margin becoming slightly longer distally, two or three long setae apically, and four short setae on inner margin (Fig. 8C–E). Vulva (Fig. 8F) rounded, projecting.

Gastric mill (Fig. 17A, C). Median tooth plate simple, without defined ridges. Urocardiac ossicle relatively broad throughout length. Propyloric ossicle semi-circular, relatively flat and broad; posterior margin curved; anterior margin with one pointed lobe medially; lateral margins slightly truncated, evenly convex (Fig. 17A). Lateral zygocardiac tooth plate with nine slender teeth, four anterior teeth largest (Fig. 17C).

#### Habitat.

**Figure 9.**
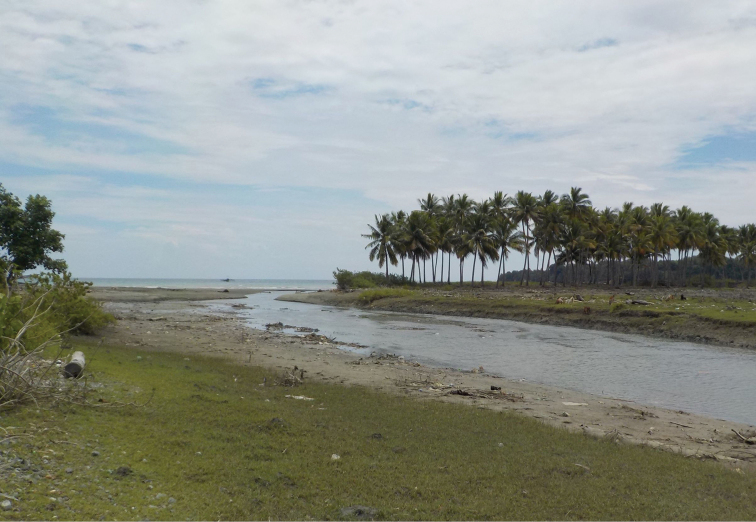
Habitat of *Tmethypocoelissimplex* sp. nov. at mouth of Towale River, Central Banawa District, Donggala, Central Sulawesi.

*Tmethypocoelissimplex* sp. nov. lives in estuarine conditions on both sandy and muddy substrata (Fig. 9). At Towale Village, it inhabits sandy substrates alongside other ocypodoids, *Austrucaannulipes* (H. Milne Edwards, 1837) and *Scopimeraintermedia* Balss, 1934, but also in muddier areas where it co-occurs with *Tubucadussumieri* (H. Milne Edwards, 1852). At Tosale Village, it was typically collected on sandy substrates. While not collected, it was also observed along a small muddy canal near local residences. It was recorded approximately 1 km further upstream beyond the estuary in non-tidal area.

#### Etymology.

The name *simplex* refers to the simple form of the cheliped dactylus that lacks a conspicuous outer subdistal dorsal projection, a character that is characteristic of other described species.

#### Remarks.

Differences to distinguish and separate the species from *T.celebensis* sp. nov. and other congeners are given under “Remarks” for *T.celebensis* sp. nov.

### ﻿ Tmethypocoelis celebensis sp. nov.

Taxon classificationAnimaliaDecapodaDotillidae

C67DE7BC-8EF7-5843-84DB-8FA4920F0285

https://zoobank.org/FA9B855D-5FE2-4007-BE15-EA27215DFD7E

Figs 10 , 11 , 12 , 13 , 14 , 15 , 16 , 17B, D

#### Material examined.

***Holotype*. Indonesia** • 1 ♂ (7.4 × 4.3 mm); Moletang River estuary, Kema Tiga, North Minahasa, North Sulawesi; 1°21'59.6"N, 125°04'38.9"E; 12 Sep. 2020; coll. DC. Murniati and D. Nurdiansyah; MZB.Cru.5574.

***Paratypes*. Indonesia** • 10 ♂ (2.8 × 1.8 – 7.2 × 4.3 mm), 8 ♀ (4.8 × 3.6 – 5.7 × 3.7 mm); Moletang River estuary, Kema Tiga, North Minahasa, North Sulawesi; 1°21'59.6"N, 125°04'38.9"E; 12 Sep. 2020; coll. DC. Murniati and D. Nurdiansyah; MZB.Cru.5180 • 10 ♂ (5.4 × 3.2 – 6.5 × 3.7 mm); Iyok Beach, East Bolang Mongondow, North Sulawesi; 0°35'06.0"N, 124°31'58.6"E; 17 Sep. 2020; coll. D. Nurdiansyah; MZB.Cru.5181 • 11 ♂ (6.3 × 3.8 – 7.9 × 4.8 mm); Tuladenggi Sibatang, Parigi Moutong, Central Sulawesi; 0°24'41.0"N, 121°07'43.9"E; 10 Jun. 2021; coll. DC. Murniati; MZB.Cru.5575 • 10 ♂ (7.3 × 3.8 – 7.4 × 4.3 mm); Maleyali, Sausu, Parigi Moutong, Central Sulawesi; 1°05'31.0"S, 120°33'39.6"E; 25 Jun. 2021; coll. DC. Murniati, Muslihun, M. Ikram; MZB.Cru.5576 • 5 ♂ (5.2 × 3.0 – 6.0 × 3.4 mm); Iyok Beach, East Bolang Mongondow, North Sulawesi; 0°35'06.0"N, 124°31'58.6"E; 17 Sep. 2020; coll. D. Nurdiansyah; ZRC 2023.0056 • 4 ♂ (6.6 × 3.8 – 7.2 × 4.1 mm); Maleyali, Sausu, Parigi Moutong, Central Sulawesi; 1°05'31.0"S, 120°33'39.6"E; 25 Jun. 2021; coll. DC. Murniati, Muslihun, M. Ikram; ZRC. 2023.0057 • 5 ♂ (4.7 × 3.0 – 6.0 × 3.7 mm); Iyok Beach, East Bolang Mongondow, North Sulawesi; 0°35'06.0"N, 124°31'58.6"E; 17 Sep. 2020; coll. D. Nurdiansyah; OMNH-Ar.12770–12774 • 4 ♂ (6.6 × 3.8 – 7.8 × 4.4 mm); Maleyali, Sausu, Parigi Moutong, Central Sulawesi; 1°05'31.0"S, 120°33'39.6"E; 25 Jun. 2021; coll. DC. Murniati, Muslihun, M. Ikram; OMNH-Ar. 12766–12769 • 4 ♂ (6.2 × 3.7 – 7.4 × 4.5 mm); Maleyali, Sausu, Parigi Moutong, Central Sulawesi; 1°05'31.0"S, 120°33'39.6"E; 25 Jun. 2021; coll. DC. Murniati, Muslihun, M. Ikram; RMNH.CRUS.D.58047 • 3 ♂ (4.3 × 3.8 – 4.9 × 3.8 mm); Tuladenggi Sibatang, Parigi Moutong, Central Sulawesi; 0°24'41.0"N, 121°07'43.9"E; 10 Jun. 2021; coll. DC. Murniati; QM W29643.

#### Comparative material.

*Tmethypocoelisliki* Murniati, Asakura, Nugroho, Hernawan & Dharmawan, 2022: Indonesia • paratypes 5 ♂ (5.3 × 3.1 mm – 5.5 × 3.2 mm); Liki Village, Sarmi District, Sarmi Municipality, Liki Island, Papua Province; 01°37'25.29"S, 138°44'26.54"E; 21 Nov. 2018; coll. DC. Murniati; MZB.Cru.5012.

#### Diagnosis.

**Figure 10.**
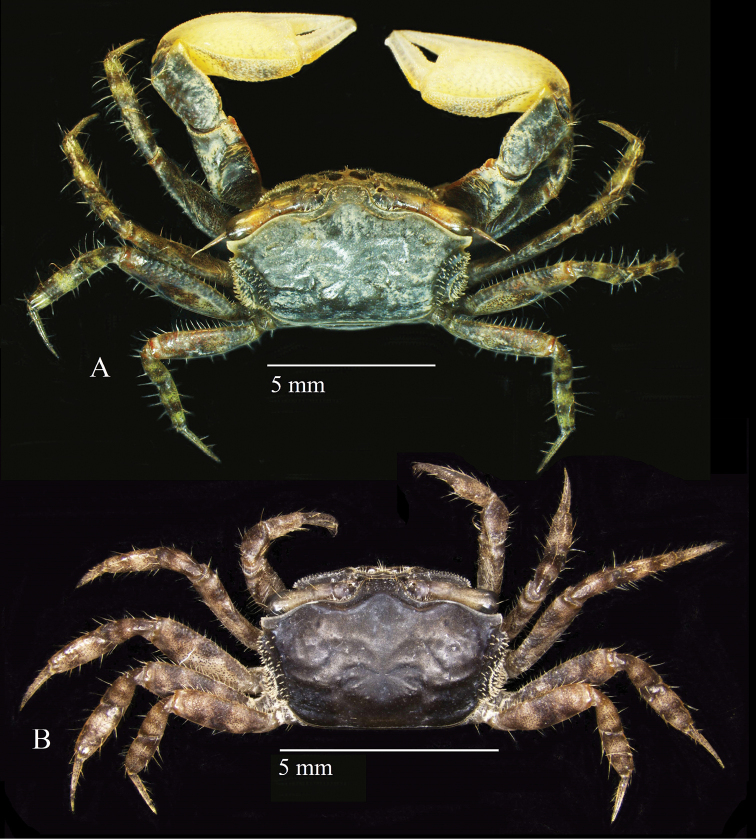
Habitus dorsal of *Tmethypocoeliscelebensis* sp. nov. Moletang River (estuary), Kema Tiga, North Minahasa, North Sulawesi **A** holotype, male (7.2 × 4.4 mm) (MZB.Cru.5574) **B** paratype, female (5.5 × 3.5 mm) (MZB.Cru.5180).

Carapace pentagonal, ca. 1.6–1.7× as wide as long (Fig. 10A). Branchial region sloping, protobranchial, mesobranchial and metabranchial regions well-defined. Sub-branchial region bulging, bearing regular setae and tubercles. Posterior margin slightly concave, ca. 0.64 distance between exorbital angles. Exorbital angle triangular, acute, directed forward (Fig. 12A). Second anterolateral tooth less acute, slightly shorter. Male pleon ca. 2.0× longer than wide (Fig. 12E). Male chelipeds long. Palm bulky, ca. 1.3× longer than wide (Fig. 13M, N). Fingers shorter than palm. Pollex short, triangular, cutting margin slightly oblique, without large differentiated tooth or lobe (Fig. 13M); cutting margin of dactylus with large teeth over proximal half, small teeth on distal half, without median lobe, upper margin with row of fine tubercles; one triangular, upturned tooth subdistally (Fig. 13M–O). G1 long, recurved, very slender; sub-proximal portion bulging (Fig. 15A, B); apical portion forming two lobes, with three short setae on outer margin, two or three long setae apically, four or five short setae on inner margin (Fig. 15C, D).

#### Description.

**Figure 11.**
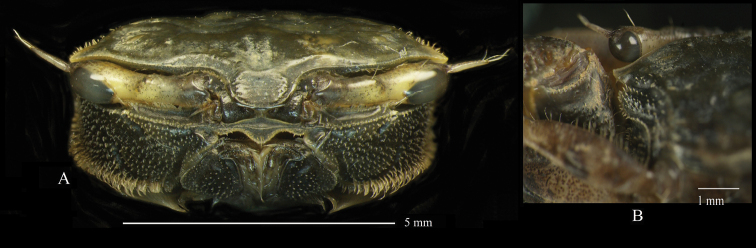
*Tmethypocoeliscelebensis* sp. nov. Holotype, male (7.2 × 4.4 mm) (MZB.Cru.5574), Moletang River (estuary), Kema Tiga, North Minahasa, North Sulawesi **A** front area **B** left merus held against external orbital angle.

**Figure 12.**
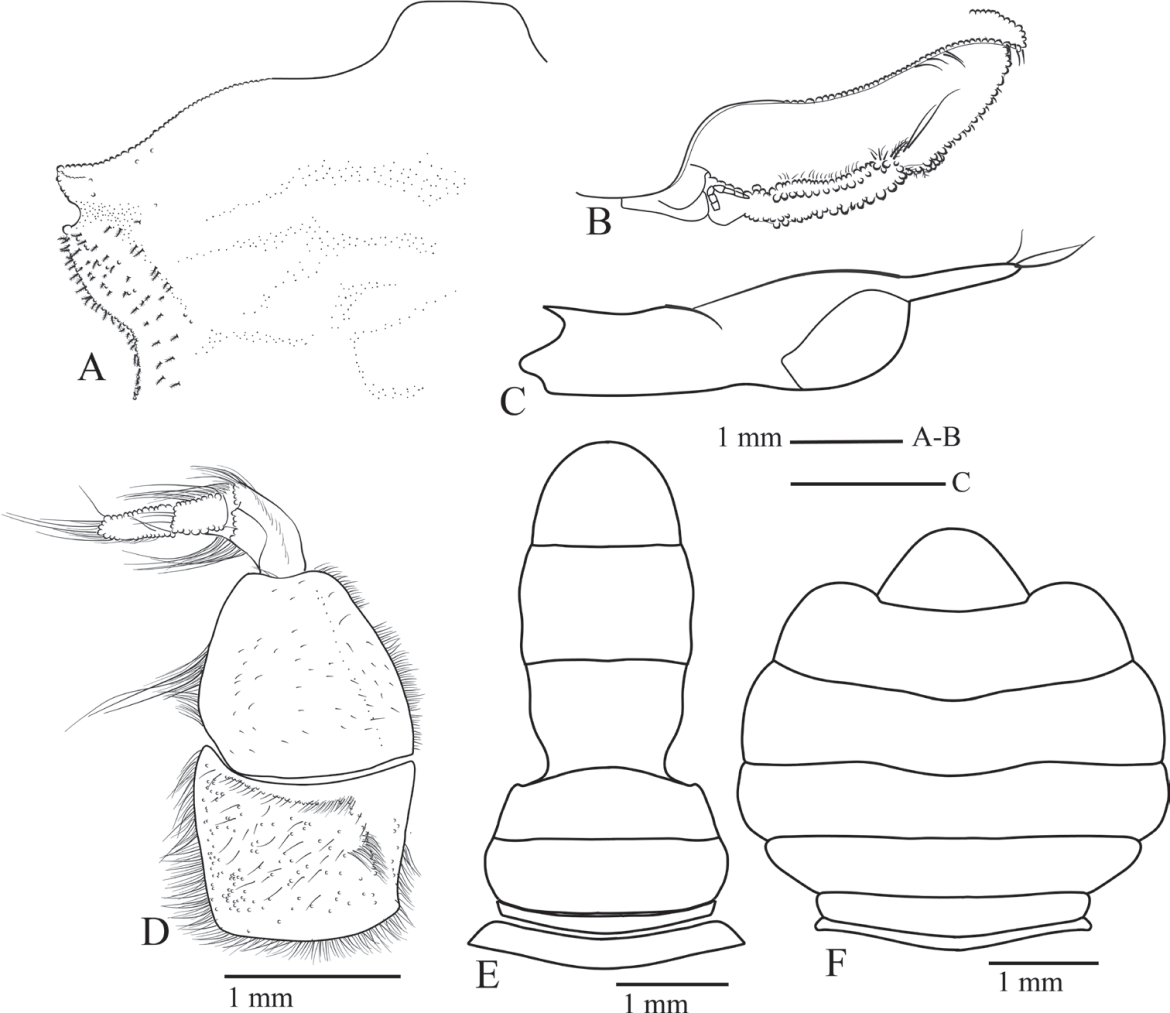
*Tmethypocoeliscelebensis* sp. nov. Holotype, male (7.2 × 4.4 mm) (MZB.Cru.5574), Moletang River (estuary), Kema Tiga, North Minahasa, North Sulawesi **A** exorbital angle **B** orbit area **C** eyestalk **D** third maxilliped **E** pleon. Paratype, female (5.5 × 3.5 mm) (MZB.Cru.5180), Moletang River (estuary), Kema Tiga, North Minahasa, North Sulawesi **F** pleon.

Carapace (Figs 10A, 12A) pentagonal; weakly convex laterally and longitudinally; ca. 1.6–1.7× wider than long. Dorsal surface smooth, regions semi-defined; epigastric lobe poorly defined. Cervical grooves, well-marked; cardiac region slightly depressed. Branchial regions sloping, protobranchial, mesobranchial and metabranchial regions well-defined. Carapace widest between exorbital angles. Sub-branchial region bulging, bearing regular setae and tubercles. Intestinal and branchial regions well-defined. Posterior margin weakly concave, ca. 0.6× distance between exorbital angles; fine ridge parallel with posterior margin forming broad rim. Lateral margin recurved with row of tubercles and short stout setae. Frontal margin rounded, moderately convergent, basal width ca. 0.19× distance between exorbital angles, ca. 0.13× at anterior margin; anterior margin with small central blunt prominence (Fig. 11A). Exorbital angle triangular, acute, directed forwardly; anterior margin with microscopic tubercles, lateral margin slightly tubercular; posteriorly followed by broad U-shaped sinus. Epibranchial tooth less acute, slightly shorter. Posterolateral facet well-defined by a crest originating anteriorly from base of exorbital angle (Fig. 12A). Supra-orbital borders sinuous, sloping backward, microscopically tubercular. Infra-orbital border with medial notch; pterygostome with inwardly directed oblique channel. Inner part of infra-orbital border with two granular ridges separated by shallow channel; upper ridge with row of setae; granules on lower ridge larger than that of upper ridge, without setation. Outer part of infra-orbital border granular, concave, ending in broad notch below exorbital angle (Fig. 12B).

Eyestalks (Figs 10, 11A, 12C) reaching exorbital angle, medial and distal diameters similar size; ocular style as long as cornea, tipped with setae; medial slope gives twisted appearance; cornea bulging.

Third maxillipeds (Fig. 12D) slightly vaulted, not completely covering buccal cavern. Ischium subquadrate, outer surface covered with spaced long setae, with one oblique row of long setae near upper margin, upper margin concave, upper-mesial angle with narrow, rounded lobe; lower-mesial angle curved; inner and lower margins with dense setae; lateral margin without setation (Fig. 12D). Merus slightly larger than ischium, ca. 1.3× longer; outer surface with regularly scattered short setae; lateral margin convex, narrower distally, covered with short setae; mesial margin straight with long setae (Fig. 12D). Carpus trihedral, subequal in length to propodus and dactylus together; mesial margin and distal portion with dense long setae (Fig. 12D). Propodus shorter than dactylus; margins entire, with long dense setae (Fig. 12D). Dactylus slender, with long dense setae laterally (Fig. 12D).

Male pleon (Fig. 12E) ca. 2.0× longer than wide. Pl1 trapezoidal, ca. 8.0× wider than long; ca. 1.3× wider than pl2. Pl2 very narrow, ca. 10× wider than long. Pl3 ca. 3× wider than long. Pl4 ca. 3.2× wider than long, lateral margins convergent distally, distolateral angle pointed. Pl5 ca. 1.5× wider than long (at widest point), markedly constricted at base. Pl6 ca. 1.5× wider than long; widest sub-distally; subequal in length to pl5. Male telson rounded, ca. 1.4× wider than long (Fig. 12E).

Female pleon (Fig. 12F) conspicuously broad. Pl1 shortest; pl2 distinctly longer, as wide as pl1; pl3 trapezoidal, longer than pl2; pl4 rectangular, slightly longer than pl3, lateral margins convex; pl5 longer than pl4; pl6 distinctly longest. Female telson (Fig. 12F) triangular.

**Figure 13.**
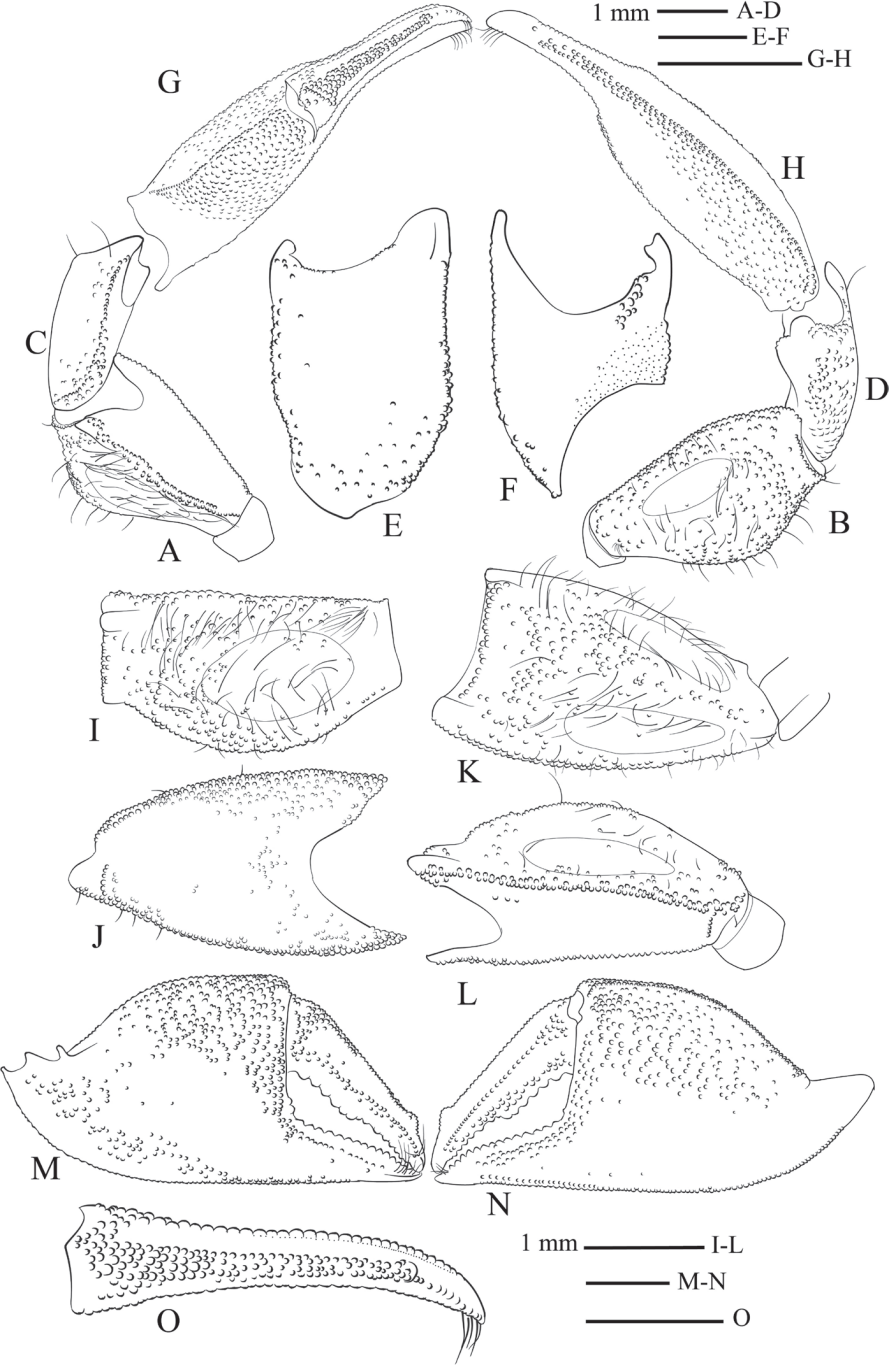
*Tmethypocoeliscelebensis* sp. nov. Holotype, male (7.2 × 4.4 mm) (MZB.Cru.5574), Moletang River (estuary), Kema Tiga, North Minahasa, North Sulawesi. Left cheliped. Merus **A** lower margin **B** outer surface. Carpus **C** upper margin **D** lower margin **E** outer surface **F** inner surface. Chela **G** upper margin **H** lower margin. Merus **I** upper surface **J** lower surface **K** upper margin **L** outer margin. Chela **M** inner surface **N** outer surface **O** dactylus upper margin.

Male chelipeds stout, long, equal. Merus triangular in cross-section; standing higher than exorbital angle (Fig. 11B); lower margin with two rows of granules extending whole length of margin (Fig. 13A); upper margin narrowing proximally, with irregular rows of granules on distal half (Fig. 13K); outer margin with one row of granules extending whole length (Fig. 13L); upper surface flattened, ovate smooth tympanum, scattered long setae around tympanum, more setation distally, microscopically tuberculate (Fig. 13I); lower surface flattened, nearly smooth, with scattered granules, lacking tympanum (Fig. 13J); outer surface convex, tympanum smaller than that of upper surface, evenly distributed granules and setae (Fig. 13B). Carpus shorter than merus, elongate, ca. 1.4× longer than wide; upper and lower margins tubercular (Fig. 13C, D); outer surface rectangular, scattered microscopic granules near upper and lower margins, median portion without microscopic tubercles (Fig. 13E); lower surface smooth, with one longitudinal row of granules, one patch of tubercles on proximal part (Fig. 13F). Palm bulky, ca. 1.3× longer than wide; inner surface granular over upper half, with granules extending over upper margin and curved to sharply cut upper margin of outer surface, distally with one row of regular granules reaching pollex, smaller granules near lower margin, median portion smooth (Fig. 13M); outer surface distinctly granular over upper half reaching to base of pollex, lower half smooth (Fig. 13N); upper margin with one row of granules, distinct groove extending below granular rows forming clear granular string (Fig. 13G); lower margin with granulation extending to lower part of inner surface (Fig. 13H). Fingers shorter than palm, lacking obvious basal gape, curved inwards, spooned-tip; cutting margins evenly serrated; inner margin at tip of both fingers with short row of stout setae. Pollex short, triangular, cutting margin slightly oblique, without large differentiated tooth or lobe, ca. 0.5× as long as palm; inner surface nearly smooth, one row of granules over proximal half (Fig. 13M); outer surface granular parallel to cutting margin, granules with similar size (Fig. 13N); lower margin granulated only along proximo-medially (Fig. 13H). Cutting margin of dactylus with teeth, larger teeth over proximal half, smaller teeth over distal half, without median lobe; inner surface with one row of granules parallel to upper margin, granulation extending from proximal to distal, one patch of granules proximally (Fig. 13M); band of granules on outer surface near cutting margin and junction to palm, one tubercular ridge extending medially parallel to upper margin (Fig. 13N); upper margin with row of tubercles terminating with triangular upturned tooth subdistally (Fig. 13O).

**Figure 14.**
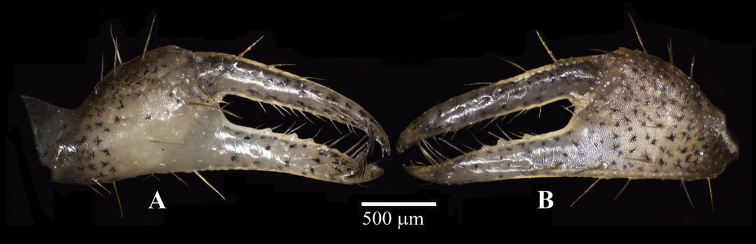
*Tmethypocoeliscelebensis* sp. nov. Paratype, female (5.5 × 3.5 mm) (MZB.Cru.5180), Moletang river (estuary), Kema Tiga, North Minahasa, North Sulawesi. Left chela **A** inner surface **B** outer surface.

Female chelipeds small dotillid type (Figs 10B, 14). Not conspicuously different from *T.simplex* sp. nov. (see description for *T.simplex* sp. nov.).

**Figure 15.**
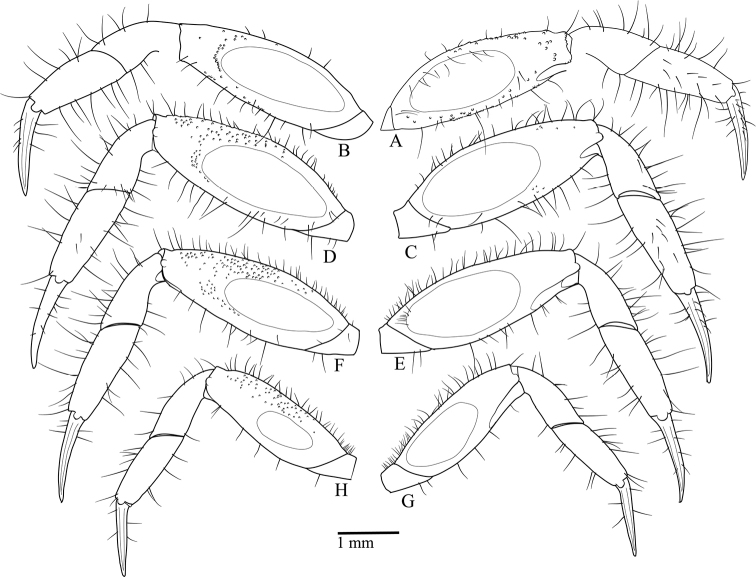
Left pereiopods of *Tmethypocoeliscelebensis* sp. nov. Holotype, male (7.2 × 4.4 mm) (MZB.Cru.5574), Moletang River (estuary), Kema Tiga, North Minahasa, North Sulawesi **A, B** P2 **C, D** P3 **E, F** P4 **G, H** P5. Right side, anterior surfaces; left side, posterior surfaces.

Pereiopods (Fig. 15) slender, elongate, P2–P5 similar; smooth ovate tympanum on anterior and posterior surfaces of meri. Tympani on posterior surfaces becoming progressively smaller from P2–P5. Dactyli nearly straight, pointed, shorter than propodi.

P2 (Fig. 15A, B) shorter than P3; merus ca. 2.7× longer than wide; anterior surface bearing scattered granules outside tympanum, granules denser near lower margin, sparse setae near lower margin; posterior surface sparsely granulate, denser distal to tympanum; upper margin convex, sparse long setae, distally tubercular; lower margin smooth, sparse setae. Carpus shorter than propodus, surfaces smooth; margins without granules, sparse setae. Propodus with anterior and posterior surfaces with few small granules only; margins with sparse long setae.

P3 (Fig. 15C, D) longest; merus ca. 2.7× longer than wide; anterior surface scarcely granular; posterior surface sparsely granulate, denser near upper margin; upper and lower margins convex; upper margin tubercular distally, sparse long setae; lower margin smooth, sparse setae. Carpus shorter than propodus, surfaces nearly smooth, sparse setae distally; margins without tubercles, sparse setae. Propodus with anterior and posterior surfaces smooth; margins with sparse long setae.

P4 (Fig. 15E, F) nearly as long as P2; merus ca. 2.6× longer than wide; anterior surface scarcely granular; posterior surface with evenly distributed granules; upper and lower margins convex; upper margin tubercular distally, spaced long setae extending whole length; lower margin smooth, sparse setae. Carpus shorter than propodus, surfaces smooth; margins smooth, sparse setae. Propodus with anterior and posterior surfaces smooth; margins with sparse long setae.

P5 (Fig. 15G, H) shortest; merus ca. 2.8× longer than wide; anterior surface without granules; posterior surface granulate, granules denser near upper margin; upper and lower margins convex; upper margin sparsely tubercular, with sparse long setae, short setae proximally; lower margin smooth, with sparse setae. Carpus shorter than propodus, surfaces smooth; margins smooth, with sparse setae. Propodus with anterior and posterior surfaces smooth; margins with sparse long setae.

**Figure 16.**
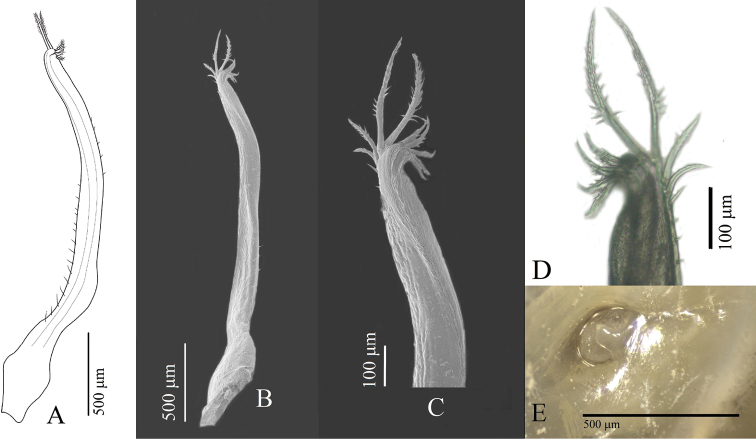
Reproductive organs of *Tmethypocoeliscelebensis***b** paratype, male (7.3 × 4.4 mm) (MZB.Cru.5180), Moletang River (estuary), Kema Tiga, North Minahasa, North Sulawesi, left G1**A** mesial view **B** dorsal view **C, D** apical portion **C** dorsal view **D** ventral view. Paratype, female (5.5 × 3.5 mm) (MZB.Cru.5180), Moletang River (estuary), Kema Tiga, North Minahasa, North Sulawesi **E** vulva.

Reproductive organs. G1 long, recurved, very slender; sub-proximal portion bulging (Fig. 16A, B); apical portion forming two lobes, with three short setae on outer margin, two or three long setae apically, four or five short setae on mesial margin (Fig. 16C, D). Vulva (Fig. 16E) rounded, projecting.

**Figure 17.**
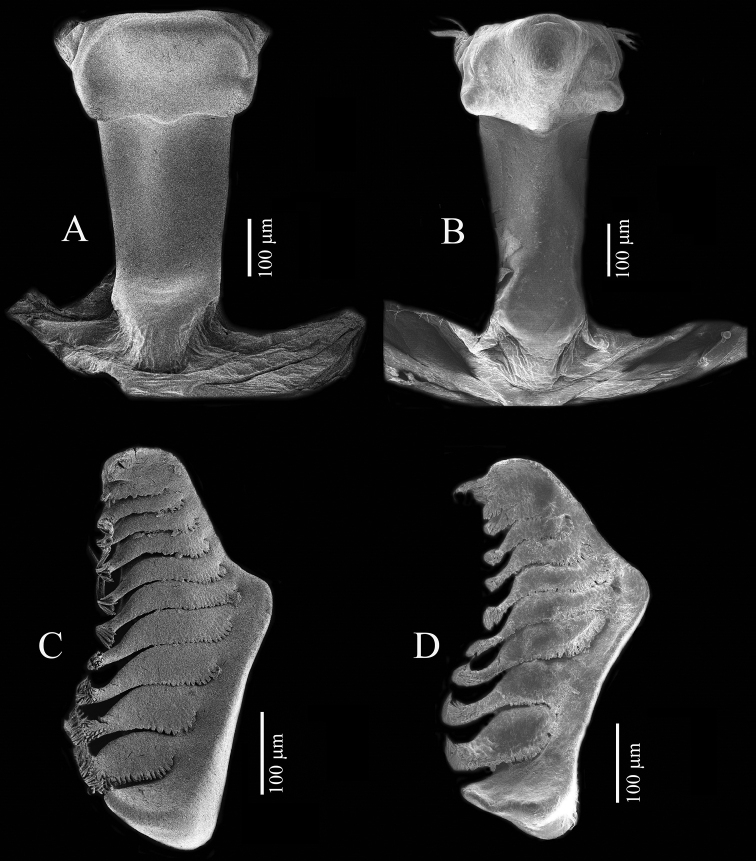
Teeth of gastric mill (posterior portion on upper part). *Tmethypocoelissimplex* sp. nov., paratype, male (6.9 × 4.0 mm) (MZB.Cru. 5183) (**A, C**). *Tmethypocoeliscelebensis* sp. nov., paratype, male (7.3 × 4.4 mm) (MZB.Cru.5180) (**B, D**). **A, B** median tooth **C, D** lateral tooth.

Gastric mill. Median tooth plate simple, without defined ridges. Urocardiac ossicle relatively narrower throughout length. Propyloric ossicle semi-circular, relatively narrow and protruding; posterior margin curved; anterior margin with one pointed lobe medially; lateral margins quadrate with anterior lobes discrete, prominent, and rounded (Fig. 17B). Lateral zygocardiac tooth plate with eight slender teeth, three anterior teeth large (Fig. 17D).

#### Habitat.

**Figure 18.**
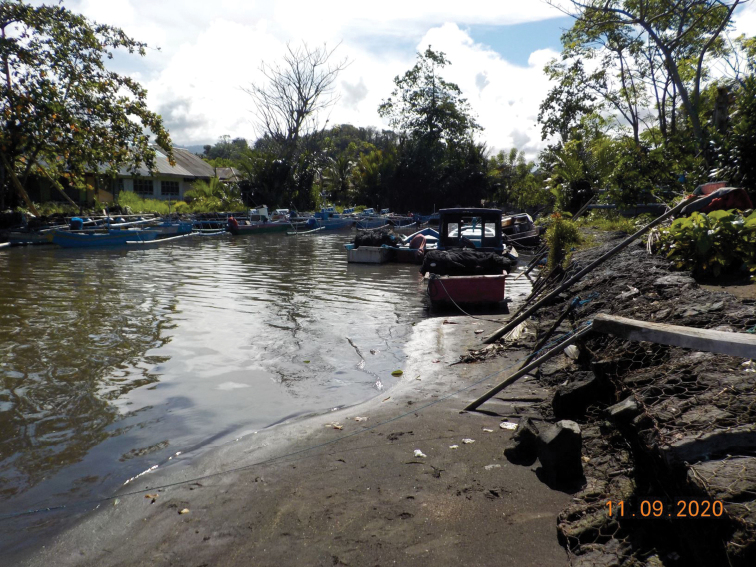
Habitat of *Tmethypocoeliscelebensis* sp. nov. at Moletang River (estuary), Kema Tiga, North Minahasa, North Sulawesi.

*Tmethypocoeliscelebensis* sp. nov. inhabits sandy substrata in estuarine areas (Fig. 18).

#### Etymology.

The species name is derived from the type locality. Celebes is the former name of Sulawesi Island, one of the great islands in Indonesia.

#### Remarks.

The two new species described here differ from each other and from the other known species by numerous characters compared below. In general, the species of *Tmethypocoelis* are all extremely similar in general morphology with only small differences in male cheliped shape and dentition (Table 1), differences in the apical setal ornamentation of the male first gonopod, and sometimes differences in the proportions of the somites of the male pleon.

**Table 1.** Comparison of male chelipeds in the species of *Tmethypocoelis*.

Species	*T.simplex* sp. nov.	*T.celebensis* sp. nov.	*T.koelbeli*	*T.liki*	*T.ceratophora*	*T.choreutes*	*T.odontodactylus*
Dactylus dorsal and outer armature	Upper margin finely tuberculate; lacking differentiated subdistal tooth; outer surface with semi-defined granular row medially; irregular granulation near cutting margin (Fig. 5M–O)	Upper margin finely tuberculate; culminating in subdistal upward, upwardly projecting triangular tooth of variable size from high and distinct to low; size not correlated with crab size; outer surface with medial granular row over entire length (Fig. 13M–O)	Dorsal band of fine granules; superior border straight, terminating in overhanging triangular tooth at about ¾ length; outer surface with 2 subregular lines of granules, superior one may extend ¾ length to tip, lower one less than that of superior	Upper margin with median row of granules, culminating into 1 prominent tooth	Finely tuberculate ridge on upper margin of dactylus terminates in outwardly directed flat triangular tooth (see Davie and Kosuge 1995: fig. 1 A, C)	Finely tubercular ridge on the upper margin continues evenly distally; outer surface has separate ridge ending in prominent triangular subdistal protrusion (see Davie and Kosuge 1995: fig. 1 B, D)	Full length medial granulate ridge; superior granulate crest terminating subdistally in strong upturned tooth (Davie 1990: fig. 3C)
Dactylus cutting margin	Evenly dentate; one wide enlarged convex tooth over proximal half	Evenly dentate, larger over proximal half, then finger narrower over distal half	Evenly dentate; smaller males with raised platform of teeth differentiated in proximal half, but less evident in mature chela	Wide and blunt irregular serrations	One small low tooth proximally	Low broad triangular convexity but lacking clearly differentiated tooth	Evenly dentate; slightly deeper medially, but without obvious differentiated tooth
Pollex cutting margin	Evenly dentate; long flat enlarged dentate tooth over most of length	Evenly dentate; straight, without differentiated tooth or lobe	Evenly dentate	Irregularly dentate	Prominent enlarged convex tooth medially	Lacking a defined tooth; slightly convex	Weakly convex; evenly dentate
Gape at base of fingers	Large	Poorly developed	Moderate	Poorly developed	Wide	Not strongly developed	Not strongly developed

A comparison of male first gonopod setation patterns of described species suggests that the possession of two or three of markedly elongated apical setae (Figs 8C–E, 16C, D; Davie 1990: fig. 2), common to both *Tmethypocoelissimplex* sp. nov. and *T.celebensis* sp. nov., is so far shared with *T.liki* from Papua and *T.koelbeli* from the Northern Territory, NW Australia. Therefore, these four species may be more closely related to each other than they are to *T.ceratophora*, *T.choreutes*, and *T.odontodactylus*, which all share a coronet of shorter more evenly sized stout setae on the tip of the G1. A more thorough analysis of relationships within the genus will be undertaken as part of a larger revision of the genus, and with the help of DNA sequencing data.

Both *Tmethypocoelissimplex* sp. nov. and *T.celebensis* sp. nov. differ significantly from *T.koelbeli* in the shape of the male pleon, with that of *T.koelbeli* being relatively narrower, and in particular Pl5 being more constricted proximally (Table 2). The pleons of *T.simplex* sp. nov. and *T.celebensis* sp. nov. are similar, however, both Pl6 and the telson are slightly proportionately wider in *T.celebensis* sp. nov.

**Table 2.** Comparison of pleonal somite proportions of *Tmethypocoelissimplex* sp. nov. and *T.celebensis* sp. nov. with the closely related *T.koelbeli* (proportions of latter taken from Davie 1990: fig. 1A).

Species	*T.koelbeli*	*T.liki*	*T.simplex* sp. nov.	*T.celebensis* sp. nov.
Pleonite 5 width/length	1.1	1.3	1.5	1.5
Pleonite 5 narrowest proximal width to distal width	0.6	0.8	0.7	0.7
Pleonite 6 width/length	1.2	1.2	1.4	1.5
Telson width/length	1.2	1.4	1.4	1.4

*Tmethypocoelissimplex* sp. nov. differs from *T.celebensis* sp. nov., *T.koelbeli*, and *T.liki* in the form and number of the apical setae of the G1. The G1 of *T.simplex* sp. nov. typically has two or three very long setae apically (Fig. 8C–E), and subapically there are three shorter stout setae on the outer margin increasing in length distally, and four short, downwardly reflexed setae on inner lobe. The G1 of *T.celebensis* sp. nov. has two or three very long recurved setae apically (Fig. 16B–D), and subapically there are also three stout setae on the outer margin, though the proximal seta is much smaller and less prominent than on *T.simplex* sp. nov., and also four or five short, downwardly reflexed setae on the inner lobe. The G1 of *T.koelbeli* similarly has two long apical setae but lacks a row of outer subapical setae and has a row of five short distally pointed setae on the inner lobe (Davie 1990: fig. 2). The G1 of *T.liki* has one long and five short apical setae (Murniati et al. 2022: fig. 20C).

## ﻿Discussion

*Tmethypocoelis* for many years included only the type species *Tmethypocoelisceratophora* (Koelbel, 1897), which was believed to be widespread from Hong Kong, China, Japan, and south to Lombok in Indonesia (Tesch 1918; Dutreix 1992; Huang et al. 1992; Murniati 2015). In recent years, however, four new species have been described (see Introduction), and the distribution of *T.koelbeli* has become more restricted (Davie 1990; Davie and Kosuge 1995; Murniati et al. 2022). Nevertheless, the genus is outwardly morphologically relatively homogenous, with only small differences among the species most obviously related to chela dactylar tooth shape, and differences in the apical setae of the G1. This is particularly exemplified by the separation of the pseudo-cryptic *T.choreutes*, that had long been confused with *T.ceratophora*, but the morphological differences were shown to correlate with the evolution of a different male courtship waving display (Davie and Kosuge 1995).

The relative morphological homogeneity within the genus is also an indication that *Tmethypocoelis* species have undergone relatively recent speciation based around small-scale biogeographic restrictions. With the complex evolving paleogeography of land-connections and sea-level changes throughout the Indo-Malaysian Archipelago over the last two million years, it can be expected that the genus may have speciated much more than previously thought. Careful collecting across a broad range of areas within the region and more careful observations of populations, including finer scale morphological investigations, behavioural analyses and genetic studies are indeed revealing this pattern, and further new species will be described by the present authors as part of ongoing revisionary work.

### ﻿General morphology

It is interesting to note that the tympani on the anterior and posterior surfaces of the pereiopods are essentially the same between the present two new species; and while the tympani are very similar on both faces of P2 and P3, on both P4 and P5 the anterior tympani are markedly smaller in size with P5 the smallest; the posterior tympani on P2 and P5 are much larger and cover a proportionately similar surface area to the first two pereiopods (Figs 7, 15). This is simply an observation, and no physiological explanation can be offered.

### ﻿Feeding morphology

Speciation in ocypodoid crabs seems to have commonly involved variations in structures related to feeding and adaptations to different sediment particle sizes or food types on which each species feeds. For example, both the setation of the second maxillipeds and the shape of the grinding plates inside the gastric mill, have proven useful in distinguishing closely related species (Davie et al. 2015). The second maxilliped has specialized “spoon shaped” setae for sorting organic matter and microorganisms from the sand (e.g., Icely and Jones 1978; Vogel 1984; Colpo and Negreiros-Fransozo 2013). Spoon-tipped setae mostly occur on the inside margins of the second maxillipeds where they hold sand grains that are then brushed by short stiff setae on the outer faces of the first maxillipeds. Such setal structures have been well studied especially in species of *Dotilla* Stimpson, 1858 and *Uca* Leach, 1848 sensu lato, and vary according to the preferred substrate particle-size composition, and the distribution of the species on the shore (Icely and Jones 1978). Murniati and Wowor (2017) were the first to use second maxilliped setation to successfully separate three species of *Tmethypocoelis* occurring in Indonesia, and to help infer micro-ecological niche separation.

Although, the second maxillipeds have not been examined as a part of the present species descriptions, the gastric mill structure has been included, and equally shows that the two new species described here have adapted to different dietary requirements (e.g., see Kosuge and Davie 2001), and this is the first study to report species-specific gastric mill differences within *Tmethypocoelis* (Fig. 17A–D). The main trunk of the urogastric ossicle is noticeably broader in *T.simplex* sp. nov. than in *T.celebensis* sp. nov.; in both species, the medial tooth plate is simple and without defined ridges. However, the lateral margins are evenly convex in *T.simplex* sp. nov., versus more quadrate margins with anterior lobes discrete, prominent, and rounded in *T.celebensis* sp. nov.; the propyloric ossicle is also flatter and broader in *T.simplex* sp. nov., versus narrower and more protruding in *T.celebensis* sp. nov. In lateral zygocardiac teeth, there are also significant differences: in particular, *T.simplex* sp. nov. has nine accessory teeth that have a broader thicker brush of apical setae than *T.celebensis* sp. nov., which has only eight accessory teeth, each with a narrower brush of apical setae. While these differences would need a more specialized study to understand the dietary implications, nevertheless, it is apparent that although *Tmethypocoelis* species are deposit feeders, the lack of fine transverse median ridges on the urocardiac ossicle (as one would find on deposit feeding ocypodids such as *Uca*; see Icely and Nott 1992) would seem to indicate that the particulate organic matter that they are consuming does not require fine grinding, and the brushes of setae and fine scales on the accessory teeth of the zygocardiac ossicles serve more of a brushing function.

### ﻿Biogeography

*Tmethypocoelis* species are essentially estuarine animals, living abundantly on estuarine mud flats and able to tolerate low salinities. Davie (1985) and Davie et al. (2010) have postulated that a short larval life in combination with local hydrological factors may be enough to lead to the allopatric separation of two geographically close taxa. Davie and Kosuge (1995) remarked that *Tmethypocoelisceratophora* and their new species *T.choreutes* are separated by the relatively narrow strait between Taiwan and the Japanese Yaeyama Islands. Davie et al. (2010) described a similar disjunction between the Chinese/Taiwanese *Mictyrisbrevidactylus* Simpson, 1858, and their new species *Mictyrisguinotae* Davie, Shih & Chan, 2010, as well as citing a number of other similar cases of closely related sibling species on either side of the aforesaid strait. They concluded that the Ryukyus appear to be much more influenced by the main Kuroshio Current in contrast to the continental coastline, which is impacted mainly by the South China Sea Current and westerly flowing Kuroshio Branch Current (Jan et al. 2002), and that the deep-water strait between Taiwan and the Yaeyama Islands plays an important additional role in the local circulation patterns of the region, so as to become an effective barrier for species that may have rapid larval development and/or abbreviated life cycles. Thus, this narrow passage of water has functioned as a barrier against genetic flow between the two regions, and allowed the allopatric speciation of sibling taxa.

**Figure 19.**
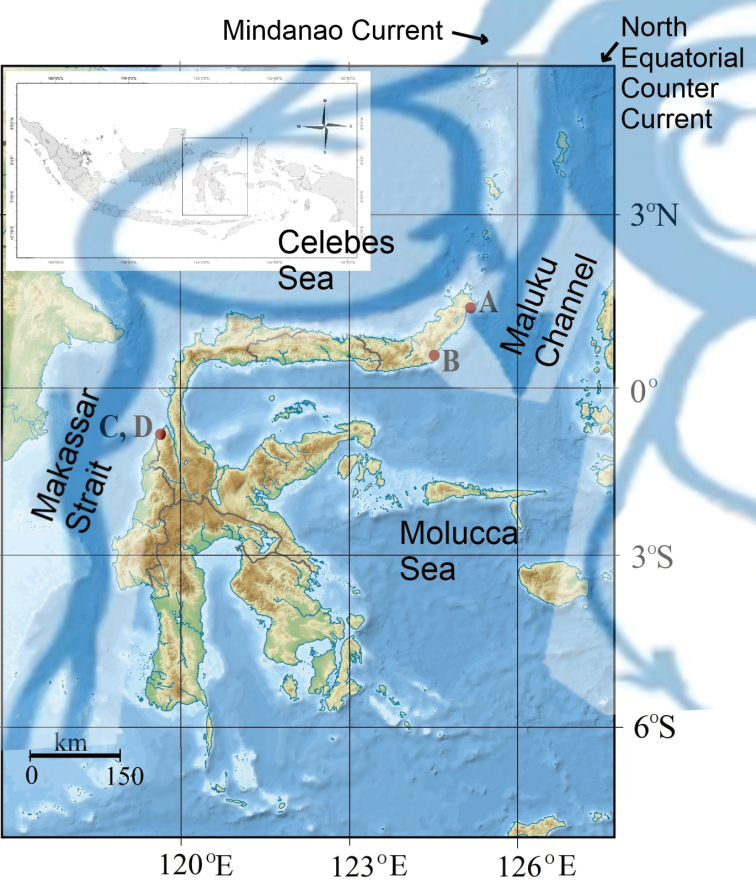
Map showing sea-current circulation patterns in the waters surrounding Sulawesi (derived from Wijeratne et al. 2018 and Sprintall et al. 2019).

In the case of the two new species described here, *T.simplex* sp. nov. and *T.celebensis* sp. nov., there is good evidence that a similar pattern of local current flow may have led to their separate evolutionary development (Fig. 19). Sprintall et al. (2019) have published an interesting map of current circulation patterns within the Indonesian Archipelago that shows the southerly flowing Mindanao current forming a counter-clockwise circulation flow within the Celebes Sea as well as flowing south through the Makassar Strait. The Celebes Sea is bordered to the east by a shallow ridge and island chain extending northwards from the tip of north Sulawesi to Mindanao in the southern Philippines; whereas the east coast of Sulawesi, home to *T.celebensis* sp. nov., is instead under the influence of an off-shoot of the North Equatorial Counter Current that flows southwards through the Maluku Channel. There are, therefore, two major separate southerly flowing current systems on either side of the island of Sulawesi, and this appears to effectively separate larval dispersal of both species to the east and west coasts, respectively.

While no genetic clock estimates have yet been applied to the two species studied here, it can be presumed that their evolutionary separation has been recent, i.e., within the last 2 million years. This time-frame has precedent in other recent speciation events that have occurred in the Indo-West Pacific region. For example, based on the molecular clock of COI mutation rates suggested by Schubart et al. (1998), Ragionieri et al. (2009, 2010, 2012) found that the sister sesarmid species *Neosarmatiumafricanum* Ragionieri, Fratini & Schubart, 2012, and *N.meinerti* (De Man, 1887) became isolated between 1.6–1.96 ± 0.34 mya (1.6% divergence); and similarly, Lai et al. (2010) estimated that the portunids *Portunusarmatus* (A. Milne-Edwards, 1861) and *P.reticulatus* (Herbst, 1799) became established around 0.78–2.5 mya, based on a 1.8% CO1 divergence.

**Figure 20.**
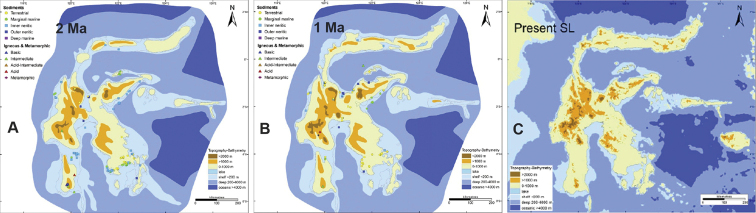
Paleogeography of Sulawesi. **A** 2 Mya **B** 1 Mya map **C** present time (after Nugraha and Hall 2018).

Given our assumption that species separation has been caused by differences in circulation patterns, then it is important to understand the geological history of the Indonesian archipelago and the geological changes that have led to the current shape of the island of Sulawesi. Nugraha and Hall (2018) have studied the Late Cenozoic palaeogeography of Sulawesi, and it is clear that it is only within the last 2 million years (since the Early Pleistocene) that, Sulawesi began to resemble its present form (Fig. 20A–C). By the Early Pleistocene, paleogeographic change across Sulawesi included the rise of high mountains and the rapid subsidence in oﬀshore basins; much of the North Arm and most of the southern South Arm appear to have emerged although the northern part of the South Arm was still a shallow marine area. Between 1.8–1.0 mya subsidence in the southern SE Arm continued, and by 1 Mya, Sulawesi was very similar in form to the present. The inter-arm basins were close to their present depths of 1.5 to 2.0 km. The North Arm was largely emergent, and there was a land connection between the North Arm and western Central Sulawesi as the Neck elevation increased. Therefore, this pattern of recent island emergence and sea basin separation supports our contention that speciation of *T.simplex* sp. nov. and *T.celebensis* sp. nov. began as a vicariant event within the last 2 million years.

Studies of survivorships of pelagic larvae under various salinity regimes, and analyses of genetic structure among different island populations throughout the Indonesian Archipelago will provide exciting insights into the speciation of coastal crabs and the evolutionary impacts of paleogeography throughout this region.

## Supplementary Material

XML Treatment for Dotillidae

XML Treatment for Tmethypocoelis simplex

XML Treatment for Tmethypocoelis celebensis
